# Attending to design when developing complex health interventions: A qualitative interview study with intervention developers and associated stakeholders

**DOI:** 10.1371/journal.pone.0223615

**Published:** 2019-10-15

**Authors:** Nikki Rousseau, Katrina M. Turner, Edward Duncan, Alicia O’Cathain, Liz Croot, Lucy Yardley, Pat Hoddinott

**Affiliations:** 1 Nursing, Midwifery and Allied Health Professions Research Unit (NMAHP-RU), University of Stirling, Stirling, United Kingdom; 2 Institute of Health and Society, Newcastle University, Newcastle upon Tyne, Newcastle upon Tyne, United Kingdom; 3 Population Health Sciences, University of Bristol, Bristol, United Kingdom; 4 School of Health and Related Research (ScHARR), University of Sheffield, Sheffield, United Kingdom; 5 Department of Psychology, University of Southampton, Southampton, United Kingdom; 6 School of Psychological Science, University of Bristol, Bristol, United Kingdom; University of Toronto, CANADA

## Abstract

**Background:**

Guidance and frameworks exist to assist those developing health interventions but may offer limited discussion of ‘design’, the part of development concerned with generating ideas for and making decisions about an intervention’s content, format and delivery. The aim of this paper is to describe and understand the views and experiences of developers and associated stakeholders in relation to how design occurs in health intervention development.

**Methods:**

Semi-structured interviews were conducted with 21 people who had developed complex interventions to improve health and/or who were relevant stakeholders (e.g. funders and publishers of intervention development work), regarding their views, experiences and approaches to intervention design. Sampling was purposive in terms of maximising diversity. A thematic inductive analysis was conducted.

**Results:**

Approaches to design varied substantially between intervention developers. This contrasted with consistency in other activities undertaken during development, such as literature review. Design also posed more challenges than other parts of development. We identified six ‘modes’ of design: informed; negotiated; structured; delegated; ‘my baby’; and creative partnership. In understanding the differences between these different modes, and the challenges posed by intervention design, we identified three key themes: enabling creativity during the design process; working with different types of knowledge; and ‘stabilising’ (developing clear shared understandings of) the intervention development to enable design.

**Conclusions:**

Design has received less attention than other activities undertaken when developing interventions to improve health. Developers take a variety of approaches to design and often find it challenging. Guidance for intervention development in health has tended to see design as proceeding in a predictable and controlled manner from acquired knowledge. Our study suggests that design rarely reflects this rational ideal. Future guidance on intervention development in healthcare should support developers to work effectively with different types of knowledge, to help design progress more smoothly and to maximise creativity.

## Introduction

The terms “design” and “development” are sometimes used interchangeably to describe the process by which an initial idea for a new intervention is progressed to the point at which there is a document or manual describing the intervention and how it should be delivered, ready for formal evaluation [1, 2]. This tendency to conflate design and development may have contributed to “design” receiving insufficient attention. “Approaches” to intervention development are guidance or frameworks which describe a method for developing interventions that others can follow [2]. A recent review of approaches to the development of health interventions identifies multiple actions within the development process [2]. These include actions concerned with understanding the nature, causes and context of a problem; identifying and summarising relevant evidence e.g. regarding the effectiveness of similar interventions; and testing prototype versions of the intervention for acceptability and feasibility. Design encompasses actions undertaken during intervention development that focus on generating ideas for, and making decisions about the content, format and delivery of an intervention. Design tends to continue throughout the development process, although there may be periods when design is the focus. It is distinct from, but interlinked with, and often dependent on, other actions undertaken within intervention development such as problem definition and prototype testing.

A complex intervention is one with multiple interacting components that requires changes in: the behaviour of those delivering or receiving the intervention; the structures and/or processes of the surrounding system [3–5]. Complexity resides not only in the intervention itself but in its interaction with systems–these interactions are likely to be hard to fully anticipate and measure, and include intended and unintended consequences. Widely cited United Kingdom Medical Research Council (MRC) guidance on the development and evaluation of complex interventions [6] has little detail on design. The key elements of the development phase identified by the MRC guidance are: 1. Identifying the evidence base; 2. Identifying/developing theory; and 3. Modelling process and outcomes. Little is said about how these might be used when designing an intervention. Although other guidance for intervention development in health does pay attention to design, particularly in relation to digital interventions [7, 8] or service developments [9, 10] to date these have been much less widely cited and used than the MRC guidance.

Reports of the development of specific new complex interventions have also tended to say little about the process of intervention design, although recently more detailed accounts have started to appear [11–13]. This relative absence of discussion of design within intervention development in healthcare is in contrast to the large volume of relevant research in fields such as product design and organisational management and innovation. Design as seen from the perspective of those working in organisational management is conceptualised within the “fuzzy front end” of new product development–the “fuzzy” recognising that this part of development is often messy and unpredictable [14, 15]. Research in this field has been concerned with understanding key features of front end activity, like how knowledge is created and mobilised; and with creativity and how teams can work to maximise the generation of useful ideas within new product development [16, 17]. Within product design, “design thinking”, has been influential. Design thinking is a collection of approaches to innovation with common features, including empathy with end-users’ needs and wants, being solution rather than problem focused, an emphasis on prototyping and using creative techniques to enable participants to maximise the number of imagined solutions [18, 19]. Design thinking has been widely used in other fields and there is increasing interest in its potential for social and health interventions [19–21].

Design thinking is closely related to the field of human or user centred design, which has its origins in computer-based technology development [22]. A recent scoping review [17] has mapped the use of human centred design in health research, identifying 21 applications in diverse geographical and clinical areas. The review authors comment that although there is considerable use of design thinking and human centred approaches within healthcare intervention development, it is difficult to bring these together in an academic literature review. This is because materials originate from different types of organisations including design agencies [23] who tend to report in the grey literature; use varied language for similar concepts and often do not report methods clearly [20]. These factors may contribute to why there has been relatively limited incorporation of this literature and thinking within academic health research.

More widely adopted within health intervention development are the approaches of co-production or co-design; the active involvement of key stakeholders–usually including the intended target population (in health terms this is often patients and the public), and those delivering the intervention [2, 24]. As with design thinking and human centred design, there are various approaches to co-design but all share a commitment to power sharing in relation to design decisions with these stakeholders. However co-design in particular, unless explicitly articulated, is a broad concept which varies in the level of involvement of stakeholders.

A qualitative study involving eleven developers of internet based public health interventions at one US university identified the importance of a strong research team, of planning and documenting decisions during design [25]. Other themes were specific to the software development context. Research which brings together learning about design from across different intervention developments to improve health is lacking. As part of a wider study on IdentifyiNg and assessing different approaches to DEveloping compleX interventions (INDEX) to develop guidance for those developing complex interventions to improve health, a qualitative interview study was conducted and is reported in this paper. INDEX also included a review of published approaches to intervention development [2], a review of primary research reporting intervention development, an e-Delphi study and a consensus workshop [26]. The aim of the qualitative study was to understand experiences and challenges of intervention development in health and healthcare from the perspectives of intervention developers and associated stakeholders in this endeavour. ‘Design’ was identified as a key focus during the conduct of early interviews. The aim of this paper is to describe and understand the views and experiences of developers and other stakeholders in relation to how design occurs in in health intervention development.

## Methods

A qualitative study involving semi-structured interviews with people who had developed complex interventions to improve health and/or who were associated stakeholders, e.g. funders and publishers of intervention development, was conducted. Ethical approval was received from University of Stirling, General University Ethics Panel (reference GUEP37) prior to commencing recruitment.

### Sampling

Sampling was purposive in terms of maximising diversity, with participants selected according to the following criteria: amount of intervention development experience; role within the development team (lead, co-investigator (including patient and public co-applicants), researcher); professional background (clinical including medical, nursing and allied health professionals; social scientists including health psychologists sociologists and economists; others e.g. product designers); geographical location (UK, other European countries, other parts of the world); and intervention type and setting. Associated stakeholders were people with a responsibility for, or who might be affected by, intervention development activity. Approached in this category were those with a role in using or implementing healthcare interventions as well as journal editors and members of research funding panels. Associated stakeholders were included to explore their views on the scope and quality of intervention development work and the approaches and methods used. An attempt was made to recruit participants whose method of intervention development had been informed by a particular approach, framework or guidance, for example, an evidence based, theory-based approach or partnership approach etc. [2]. However, at interview this classification was difficult to apply because participants had used varied approaches in different intervention developments and sometimes deviated significantly from their intended approach. The aim of our purposive sampling strategy was to understand design from multiple perspectives, rather than to compare the experience of specific subgroups.

Potential participants were identified via a variety of sources: authors of papers identified via the two INDEX reviews [2, 26], members of the INDEX project international expert panel, our personal knowledge of individuals working in health services research and on funding panels, funding websites; snowball sampling from initial participants (e.g. in an attempt to increase international participation). Sampling was also informed by the developing analysis, for example we recruited a participant with an art and design background, in response to the developing theme around creativity. During fieldwork, the team monitored the characteristics of participants to ensure maximum variety within the sample.

### Data collection

Potential participants were approached by email, provided with an information sheet and invited to complete a brief questionnaire. The questionnaire was personalised, referring to a specific intervention development project that the potential participant had been involved in. Projects were identified either from the INDEX review of primary research studies, or via internet searches. Respondents were asked whether they had participated in other intervention development which used a different approach. Respondents were also invited to comment on key challenges for intervention development and to suggest other potential interviewees. Associated stakeholders were sent a modified questionnaire, asking whether they had personal experience of intervention development in addition to their role (e.g. funding panel, editor). Questionnaires provided useful background to the interview, enabling optimum use of interview time, but participants were not included or excluded on the basis of responses given. Interested participants were then contacted by NR to arrange a suitable time and mode (skype, telephone, face-to-face) of interview. Prior to interview, NR familiarised herself with the interviewee’s published intervention development work. Before interviews, participants completed a consent process (verbal for telephone/skype and written for face-to-face). All interviews were conducted in English by NR, an experienced qualitative researcher.

A topic guide was developed and agreed upon within the team covering aims, participants, actions taken and outcomes from intervention development, including what aspects had been challenging and why, and what had worked well. Some interviews took a broadly narrative approach, with interviewees describing the process of developing a single intervention from initial idea through to evaluation and implementation, with a focus on any aspects that had been challenging and how those challenges were resolved. Other interviewees gave examples from, and discussed their experiences of, more than one intervention development. All interviews were digitally-recorded using an encrypted audio recorder. Reflective field notes were made after the interview, to assist with analysis and to record any other information not gathered during the recorded interview. Interviews were transcribed verbatim, checked and anonymised. In some cases, email correspondence took place following the interview to clarify discussion points. These, and completed responses to the email recruitment questionnaire, were included in the coded data set.

Maintaining anonymity of the participants was particularly important as some participants were well known within the health research community. The need for this was confirmed during early interviews when one person reviewed their transcript and asked for a small amount of text to be deleted and another person asked during the interview for a specific section to be considered “off the record”. Following these interviews the team took the decision that only NR would know who had been interviewed. To ensure anonymity, identifying details were removed by NR before transcripts were shared with the research team.

### Analysis

Data analysis broadly followed a thematic approach, including data familiarisation, coding, searching for and defining themes [27] and included data saturation [28]. Data collection and analysis occurred concurrently; emergent themes and issues from earlier interviews informed the content of subsequent interviews and the topic guide evolved throughout data collection. As data collection and analysis progressed, a coding frame was devised, tested and refined by NR (social scientist), PH (academic general practitioner), KT (social scientist) and ED (academic allied health professional), and applied to the transcripts by NR using NVivo 11 [29]. Each member of the team applied the developed coding frame independently to one transcript; the aim of this exercise was to allow comparison of, and reflection on, differences. The emerging analysis was discussed at regular team meetings (NR, PH, KT and ED) and with AOC. Data collection ceased when no new themes were identified or significantly elaborated i.e. “saturation” [28].

In this paper, quotations are used to illustrate findings. Some minor details have been changed, for example gender, to reduce the chance of others identifying the participants. Interviewees often used the terms ‘design’ and ‘development’ interchangeably during the interviews. In the quotations that follow there are a number of places where interviewees refer to ‘development’ when describing ‘design’. The original wording has been kept.

## Findings

### Description of participants

The interviews were conducted between February 2017 and January 2018. Twenty-nine individuals were invited for interview and 21 agreed (Table 1). Most of those who declined to be interviewed were based outside the UK. Most participants had held various roles across different intervention developments (e.g. lead on one, co-investigator on another). Where this was the case, they were categorised either according to their role on the intervention development most discussed; or, if no single intervention dominated discussion, to their most senior role within an intervention development team (i.e. lead: co-investigator: wider team). Only two participants (both on research funding panels) had no direct experience of developing interventions. Most of those characterised as developers also had experience of one or more stakeholder roles; a clinician or patient using health interventions, roles on funding panels and/or as journal editors. Between them, participants had experience of a wide range of intervention development approaches, including theory-led, participatory, target population centred and combined approaches [2]. Seventeen participants were based in the UK (including three who had previously lived and worked in other countries); two in other European countries, and two in North America. Seventeen of the interviews were conducted by telephone, two by Skype (with video), and two (both with patient and public contributors) face-to-face. Interview duration varied from half an hour to one hour 42 minutes with an average of 60 minutes.

**Table 1:** Participants’ characteristics (n = 21).

	Number
*Discipline*	
Clinician (doctors, nurses, allied health, public health)	10
Methodologist (health psychologists; health economists; sociologists, product design)	9
Patient	2
*Role in intervention development*	
Senior leads (had led multiple intervention development projects	4
Project leads (had led one or two intervention developments)	4
Co-investigators^1^	8
Wider team (Contract researchers^2^ and stakeholders^3^)	3
No personal experience of development (funders of development)	2
*Country*	
UK	17
EU	2
North America	2
*Gender*	
Female	13
Male	8

^1^Co-investigators were people who were named on the funding application and were typically involved in all stages of the intervention development project from conception to completion. They shared the responsibility for the project with a project lead and were core members of the team. Patient and public contributors were included in this category if they were named on a project proposal or funding application.

^2^Contract researchers were employed to carry out tasks associated with the intervention development. They typically knew the detail of the intervention development process, but were not involved in the conception of the project.

^3^Stakeholders were health professional, patient or public contributors who would have a role in using, purchasing or implementing the developed intervention. They might be consulted about aspects of the project and the plans for the proposed intervention, but did not have a responsibility for the successful delivery of the intervention development project.

### Design is more varied and more challenging than other intervention development actions

The intervention development processes discussed varied widely in terms of type and setting of intervention, team configuration and approach taken. Yet most interviewees described undertaking similar actions in similar ways. These included literature reviews, exploratory qualitative and/or quantitative data collection to understand the health problem and its context, and early feasibility and acceptability testing. These actions usually presented interviewees with few difficulties. Where there were challenges, interviewees described being able to respond to them, for example in handling the quantity and diversity of published literature, or in recruiting sites to participate in early feasibility testing. In contrast, the action of designing the intervention, that is generating ideas and making decisions about the content, format and delivery of the intervention, varied substantially, and challenges were more difficult to overcome.

### Six modes of intervention design

From the processes and actions described by interviewees, the team identified six modes of intervention design. These modes are not intended to be an exhaustive taxonomy of design approaches but more an illustration of how people go about it and the issues raised. In the first three modes—delegated design, creative partnership and negotiated design—design took place beyond the core intervention development team. In the latter three—informed design, structured design, and ‘my baby’ design—the creation of the intervention largely took place within the development team. Some interventions operated in one mode for the early stages of development, moving into a different mode later in the process. For example, the programme theory or logic model might be developed in ‘informed’ design mode and operationalised in ‘negotiated’ design mode. These six modes are described below.

#### Delegated design

Here, part of the design process was delegated to an external expert collaborator, developer or provider of similar interventions. Examples included web designers and providers of training interventions for healthcare professionals. In some cases, the development team presented this design expert with a very detailed brief. In other cases, they presented an outline of the intervention which the designer then used to create a prototype. One example was a web-based intervention where the team specified different modules that the website must contain, as well as information about how they wanted users to be guided through the website, but other aspects of how the website looked and worked were left to the external expert. Although sometimes the development team and design team remained separate, in other examples the roles between the research and design teams were more fluid. For example, expert designers would attend or lead stakeholder workshops and occasionally become involved in qualitative data collection. Teams that delegated the design did so because they recognised that external experts might have specific expertise and insights in relation to design and context:

*So I had all the information which came out saying you need to have this this this and this in your intervention and then what I did was I took it to an NHS person, someone who knows how the […] intervention would look to give me a prototype…So the elements of it came from the interview work and how it actually looked was someone in the NHS who could make it work*. *INDEX13 –clinician, project lead, associated stakeholder*

*We contracted a couple of people who were very experienced in developing this sort of intervention, to develop this new intervention. But to help them do that we fed them the results of the consensus conference and in fact one of them was actually present at the consensus meeting as well… I mean there’s several reasons for working with groups like [expert collaborator]. One is that they’ve got the real expertise. But two is because they’re outward facing into the world of service provision*. *INDEX16 clinician, senior lead, associated stakeholder*

#### Creative partnership

This involved working together with stakeholders with the aim of maximising creativity and the generation of new ideas. Here, the team included someone with specific expertise in the process of design, often drawing on ideas from out with the field of health. Teams in this mode used games and exercises to encourage creative thinking.

*So an example was, and this was at a very early workshop, they split people into three groups and we all had baseball caps to put on [laugh]. So one group was Team [tech company], one group was Team [supermarket] and the other group was the [military services], and then each group was tasked with 'if you had [a complex intervention], what would it look like and how would it be delivered?' And so they went into a completely different mode of thinking, and it just meant that people got away from the constraints and systems and all that stuff that stops you thinking creatively, but rather than just saying 'oh let's do blue sky thinking' it was better than that because it gave you a different hat to put on, literally, hat to put on and to think about it from a different angle. INDEX01 methodologist*, *co-applicant*

#### Negotiated design

This often happened when design included working out and negotiating the operational detail of the intervention with the people who would deliver it in practice. In these cases, the core elements of the intervention might have been decided previously. The stakeholders here were typically very expert and experienced in the context in which the intervention would be used. A key difference between this and creative partnership was the extent to which the agreed design was intended for use beyond those participants involved in the design process. In negotiated design, the primary focus was on the current team and setting, and the expectation was that the intervention would need to go through a similar negotiation process before delivery in other settings. There appeared to be a bargaining process–between the development team’s vision of what the intervention should be, and the delivery team’s limits of the changes they were prepared to make to deliver it. Rather than a generalizable intervention, the primary aim of the negotiated design process was the acceptance by the delivery team of a specific version of the intervention.

*So that’s why we took a lot of time to translate our programme theory together with our local stakeholders into the feasible programme which was adapted to the local context […] to translate it in to something that works within the local context of our hospital*. *INDEX23 clinician, project lead*

#### Informed design

This was a very common design mode. It shared some similarities with creative partnership, frequently involving a series of stakeholder engagements such as workshops. A key difference was that the stakeholder engagement was used by the team as a source of information that was then considered alongside other sources of knowledge when the team generated the intervention components. Teams operating in this mode tended to focus more on explicit knowledge–“information”–while they did seek the experiential knowledge of stakeholders this tended to be converted into explicit knowledge (e.g. via conduct and analysis of qualitative interviews) and then considered as a resource alongside other evidence. Idea generation and decision making typically happened at team meetings rather than in conjunction with external stakeholders. A second difference was that although there was occasional mention of ideas, design was presented much more as decision making, with the intervention appearing to emerge from the collated information from different sources. The example from INDEX28 below is typical of this design–the stakeholder group was used to sense check the emerging design but key decisions happened elsewhere.

*So we drew all the resources, I guess in a sense triangulated all of that information, and came up with kind of a straw man of what the intervention looked like….we presented all the information at the workshop to see how it resonated with people*. *INDEX28 clinician, senior lead*

#### Structured design

Here the design was framed by an external pre-existing structure such as a theory or framework. Design consisted of working through a pre-existing framework, matching or identifying intervention components to fit with the structure.

*If you take something like [theory], what it allows you to do is think broadly, that you're not missing anything. So, when we all sit in a room, whatever shape or size of the group, the purpose is to have a structured discussion where we're covering all the bases, we're not just going ‘what’s foremost in our heads’. So, that's all it does in terms of prompt, right, we've considered what’s going on in the individual, we have considered psychologically, emotionally, we are considering the external environment, social environment and the physical environment … and you can apply that, at stakeholder meetings, apply it in any arena*. *INDEX27 methodologist, co-applicant*

Similar approaches can be found in the literature (e.g. Band et al [8] whose HOME BP intervention was designed with reference to the Behaviour Change Wheel and Normalisation Process Theory [30, 31]). Structured approaches like this have the advantage that the origin of intervention components is relatively clear and replicable. However, some interviewees described intending to apply a specific theory but not adhering to it because they had found it constraining and inflexible in practice.

*When we wrote the initial grant, [behaviour change theory] was absolutely an essential, but…how we actually used it wasn’t how we originally thought… so we thought we would take a cook book off the shelf and then apply it and this would tell us what to do next… And we went through so many sessions and it just wasn’t actually helping. And with the most respect to [health psychologist within team], [they] accepted that. It was telling us what we were doing, it was telling us where what we were doing fitted into the behaviour change theory in literature, but the [behaviour change theory] was not telling us where we should go next*. *INDEX20 clinician, senior lead*

#### My baby

There was only one example of this entrepreneurial style in the sample, where almost all the initial intervention design was conducted by one person, although stakeholders became involved later when refining the intervention.

*The pathway I made rather paternalistically was my baby and I didn't have [patient and public involvement] I'm embarrassed to say, I didn't involve primary care*. *INDEX30 clinician, project lead*

Although there had been a lack of stakeholder engagement during design, this did not appear to have led to poor acceptability and feasibility and the interviewee was able to describe how this intervention has been widely accepted and implemented in routine healthcare. This may have been because the individual described how the intervention was based on years of thinking about the problem and informal discussions with colleagues, as well as a literature review and data collection to understand the context.

### Three challenges for intervention design

In understanding the differences between these six design modes, and the challenges posed by intervention design, we identified three key themes: “stabilising” (developing clear shared understandings of) the intervention development to enable design; working with different types of knowledge; and recognising and enabling creativity.

### Stabilising the development to enable design

Participants often described a period during which the intervention development process became unclear, stalled or appeared to go round in circles. This could take place at any point during development, and teams described a situation that felt chaotic and messy and that needed to “stabilise” before further design could take place. The size and diversity of intervention development teams could lead to instability, as could a lack of clear leadership and direction:

*There are more people maybe with their own agendas now so it's not just the [original focus] anymore, and if it's going out to all these different areas then … which is fine, but we've got to, I don't know what we've got to do, I think it's because I don't know where we're going or exactly what we're doing*. *INDEX31 patient co-applicant*

Teams were often unprepared for this period of instability and, in attempting to move forwards, the actions taken could generate further problems. Faced with a lack of consensus, some people reduced the size of their decision-making team. Although this might reduce the immediate challenge of conflicting perspectives, this approach risked reducing the quality of the design, if the smaller team lacked key skills.

*I think the issue has been that I thought [designer’s] job was to facilitate the development of the intervention. But the way they went about it was unacceptable to the PI [project lead]–largely because of the differences in personality and working styles. So [project lead] then decided that a smaller group… would develop the intervention. The issue–for me- has been that I don’t think we have had anyone with the skills needed to do this in that smaller group. So we had endless painful meetings… it just felt like we wasted months on an incredibly frustrating process*. *INDEX01 email follow up to interview methodologist, co-applicant*

For some teams, using a structure or theory (as in the “structured design” mode discussed earlier) was helpful in facilitating the design process, because it provided a framework to work through. However more commonly within our sample it was not helpful in the way expected, because the framework did not seem to match the reality of the intervention context.

*We were trying to apply a model which was very neat, to a situation that was very messy, it seemed to me*. *INDEX18 clinician, senior lead*

Stabilisation was achieved when there was a clear, shared understanding of the nature of the problem and the direction of travel in terms of potential solutions. Sometimes teams could pinpoint a specific stabilising factor that helped them to do this. As with the design of the intervention, some interviewees described how the solution to dealing with the impasse came as a sudden insight or a flash of inspiration.

*There was a bit where that was all a bit wibbly and big pieces of paper, and then we had what we call a lightbulb moment, and then it came to us, in what we call the grid*. *And the information was received into the grid, and there you could start to really see ‘we know this, we know this, oh, and we know we don’t know this’ INDEX20 methodologist, co-applicant*

Conceptualising the intervention in a visual way seemed to help with stabilising the intervention. In a similar manner to the “grid”; INDEX29 described drawing circles to see how bits of the intervention connected to other parts. Developing and returning to logic and conceptual models could also fulfil a similar role.

Key individuals could also act as stabilising factors; providing a clear sense of direction and purpose. INDEX02 for example talked about how a designer acted as a guide during the design phase, providing a stabilising structure (although designers could destabilise if other key team members were not on board with this working style as in the earlier quote from INDEX01).

*[the funding application] was very vague as to how the intervention would be developed, it said using co-design methods, or might have said using participatory methods. But then when we got to that stage [project lead] got back in touch with [designer] and so [designer] was involved right from the beginning of that intervention development stage and helped us structure it really*. *INDEX02 methodologist, contract researcher*

### Working with different kinds of knowledge

Development typically started, as previously noted, with a phase of knowledge generation, including formal literature reviews. Design required developers to work with this knowledge and here problems sometimes arose. Knowledge was rarely available in a consistent, comparable and directly applicable form–it included formal, explicit knowledge, such as findings of literature reviews, and more tacit, experiential knowledge such as the views and insights of stakeholders about the likely acceptability and workability of the intervention.

This presented two linked challenges for the development team. The first of these was how to proceed when knowledge from different sources conflicted. Teams in this situation had to decide whether, for example, to give greater priority to the findings of the literature review or the views of stakeholders–and how to compare these very different types of knowledge.

*I think the thing that was most interesting to me or most helped me was things like the workshop and the meeting and gaining other people's perspectives because although results from a survey and stats stuff can tell you something, I think people's opinions were really important in the process*. *INDEX08 methodologist, contract researcher*

*I think it is very important to find the hole in the knowledge and to have a robust understanding, and a depth of understanding in the area that you’re trying to develop an intervention in*, *but also being flexible about the literature review. And being practical about bringing in your clinical expertise into that fold and giving that credence as well. INDEX09 clinician, project lead*

Underpinning intervention development were implicit and explicit aims and values [32]. Different forms and sources of knowledge (findings of a systematic review; qualitative research, team and stakeholder views) might be valued differently. The relative priority given to different sources of knowledge varied between teams, was unpredictable and could change during design–for example stakeholders’ tacit knowledge could overturn even strong prior evidence:

*I made a marvellous presentation to the [patient and public involvement] Group about why we were not going to work with families, because part of the review suggested it wasn’t necessary, part of the checking with other colleagues we’d done, said it would be far too expensive, blah de blah. Everything on the table said it was a bad idea*. *They totally turned our minds around. Because they knew, they argued back about how it felt, how it was different. INDEX20 methodologist, co-applicant*

The second area of challenge when working with different types of knowledge was translation. This refers to the movement and making sense of ideas and information between different elements of the design process, e.g. from a literature review, to design workshops; from a design workshop into a team meeting to decide the prototype design. Knowledge generated during development, for example from a systematic review or qualitative research could be difficult to apply in design:

*So everybody says 'oh yeah we're going to do … we'll do some [qualitative research] and then we'll do some task groups and then we'll have our intervention' and so I think people have an idea of what the process should consist of but I think what people don't understand is the real nitty gritty of that*. *So how you turn, so like with our five themes, I think that's quite a good example, how do you go from five themes to an intervention? What's the guidance on that? INDEX01 methodologist, co-applicant*

To translate their knowledge into a usable form, some teams created “evidence statements”, which summarised key findings from the evidence review, thought to be relevant to the design. However, the language used within the evidence statement might not be accessible to all intended audiences. One potential solution was to further translate the statements into a visual form.

*In that first workshop we had visual graphics put on the walls around the room in which we had the workshop going on… so it was whatever the evidence statements were in a summarised way just as visual triggers around the room*. *INDEX02. Methodologist, contract researcher*

Teams often anticipated the need to translate academic findings into a non-technical format for stakeholders, but translation was also an issue between different parts of the team or network of collaborators.

*We definitely found in terms of language and use of terminology, a lot of the disagreements and the tensions that were going on were more that people weren't understanding what the others were saying, rather than they completely disagreed—sometimes if it was on email people wouldn't be in agreement but then if we all sat down together and were able to explain it, we realised everyone was trying to say the same thing but using different terms, different language*. *INDEX02 methodologist, contract researcher*

A drawback of translation was that it usually involved a filtering process–someone–usually one or more team members–had to decide what information was relevant and appropriate to present. Thus the translation process privileged certain perspectives. Some teams deliberately avoided the challenge of translation, as here where INDEX29 describes preferring stakeholder input not to be influenced by the perceptions of the research team:

*We gave them [stakeholders] very little information. We didn't want to tell them how we thought it was going to work, we wanted them to tell us how they thought it would work*. *INDEX29 clinician, senior lead*

### Recognising and enabling creativity

The role of creativity, in terms of where ideas came from about the intervention, and how these ideas were generated, was often unclear and under-acknowledged. Developers could find it hard to articulate how the ideas for their intervention had come about.

*RES: So on that project I think … I mean, I don't know because it's still like “and then the magic happened”*.

*INT*: *It's the magic bit I'm trying to …*

*RES: I know it is but I don't think I can really unpick it. INDEX01 methodologist*, *co-applicant*

Developers operating in the creative partnership mode were most likely to pay attention to maximising creativity within the design process.

*It's really just about the kinds of tools and activities and the questions that I would use to support people to come up with ideas. INDEX07 methodologist*, *wider team*

*We had different groups, so patients, volunteers, healthcare providers, community organisations, and within each of the groups there would be smaller groups of two or three people that would then come up with their persona. So it wouldn't be their own perspective […]so then the conversation emerged*, *well how would they find out about a programme like this…who would they talk to, what kind of policies are in place…So they really got into the nitty gritty nuts and bolts of the intervention to talk about all of this but through the eyes of their persona…And when we added up all the things that we got from the groups…, we found that we had probably thought about half of the things beforehand and the other half were things we hadn't considered. INDEX29 clinician, senior lead*

The ‘my baby’ example also talked a lot about ideas

*There must be countless clinicians like me who have an idea and don't know where to slot it in*, *and does the NHS, do we need a portfolio of ideas, an ideas forum? INDEX30 clinician, project lead*

Developers working in other design modes tended to talk a lot less about where and how the ideas for their interventions arose. Design was discussed instead as a set of decisions made within meetings, without reference to ideas and creativity. This relative absence of creativity from descriptions of the development of interventions may be because ideas do not arise in a neat, systematic or replicable way.

*It jars a little bit with the language of health intervention development …the way that other disciplines or social science would describe what we do… you know, it's very much getting a replicable process and a rigorous one and I think it's really interesting to me the language that's used to describe it because I think what design brings to that*, *that isn't there is the acknowledgment that, creativity it needs space and you need to inspire people's imaginations a little bit and tap into that. INDEX07 methodologist, wider team*

The six modes that we identified differed in how they related to these three challenges of intervention development. Table 2 outlines features of the six design modes in relation to these three themes.

**Table 2:** Modes of intervention design and themes relating to the challenges of the intervention design process.

	Stabilising	Working with Knowledge	Creativity
Delegated mode	Delegation usually requires a clear brief for design. Strong relevant experience stabilises process. Problems can arise if design team move too far from solutions envisaged by team.	Mainly practical wisdom, craftsmanship–incorporates scientific element in design brief.	Design team have strong practical and technical contextual knowledge increasing creativity–but potential solutions may be limited to those within designers’ experience/expertise.
Negotiated mode	Operationalising core design features and/or programme theory [33] provides a clear objective as long as there is a shared view of desired endpoint. Participants work together towards a “win-win” outcome.	Focus is on practical, context specific knowledge. Scientific element is incorporated in programme theory.	Creative solutions to challenge of operationalisation. Mobilise practical knowledge to find solutions.
Creative partnership mode	Clear design process can provide stability–but this may be dependent on the skills of those individual designers involved, and the preparedness of other team members to engage with unfamiliar approaches.	Often focus is on practical and social knowledge. Teams can struggle with how to incorporate scientific knowledge.	Strong focus on creating environment which is enabling of ideation.
Informed mode	Often focus on defining the problem–less solution focused.	Most likely to rely on formal and scientific knowledge–access to tacit knowledge more likely to be via more. formal methods such as qualitative research.	Focus on knowledge in form which may be less likely to generate ideas (formal rather than active and experiential). Ideation may be limited, unacknowledged and occurs within team.
“My baby” mode	Solution-focused–individual has a clear sense of direction–but may struggle to take forward unless able to engage others in their vision.	Practical and explicit knowledge resident in one person (or small team). May suffer from less systematic consideration of accessing tacit knowledge of all relevant stakeholders.	Often driven by a creative idea but may need support to creatively fill in the detail.
Structured mode	Structure may be helpful if simple and a good fit–but can lead to cognitive overload, and structure may not help with solution definition.	Depends on the nature of the structure—where theories formed the structure this could lead to an emphasis on scientific knowledge, and less attention to accessing tacit knowledge of stakeholders.	Structure may restrict the design process.

## Discussion

There was considerable variation in how developers approached design and this was an element of intervention development which could be challenging. In seeking to understand this variation we have characterised six different modes of design, which vary in terms of how ideas are generated and decisions are made. We also identified three key themes: stabilising the development to enable design; working with different kinds of knowledge and recognising and enabling creativity. These themes relate to important differences between the modes and the challenges presented when integrating design with research methods.

Our inductive theme, “stabilising the development to enable design” has links with the concept of equivocality, *“the existence of multiple and conflicting interpretations among project participants”* p553 [34]. Frishammar highlights that design teams tend to focus on a reduction of uncertainty–with information generating activities. Less attention may be paid to the reduction of equivocality, although both are essential to successful project completion. Whereas uncertainty reduction activities focus on collecting and analysing information, equivocality reduction requires discussion and work to achieve consensus and shared understanding. As with the intervention developers that we spoke to who generally had considerable expertise and experience of reviewing literature, the engineers in Frishammar’s research knew how to address uncertainty, but struggled more with equivocality.

In working to reduce instability/equivocality, expressing the intervention visually and working together to create a logic model appeared to be helpful. This finding supports that of de Silva et al [35] who found that developing a Theory of Change together facilitated stakeholder involvement in design. Objects and representations may be helpful in co-ordinating work activity [36] particularly when these act as “boundary objects”–creating a bridge between different social worlds (for example between academics and practitioners or between patients and clinicians) [37]. By contrast, for some teams, attempting to use an existing theory to structure their design appeared to increase equivocality. This may be because attempting to adhere to a structured approach has a “cognitive cost” which adds to the “cognitive load” of design [38].

We identified that intervention design involved working with different types of knowledge; formal or explicit knowledge such as the results of a literature review and tacit knowledge–such as the practical wisdom of stakeholders. A challenge that came across clearly in our data is that often this knowledge is conflicting, there may be gaps, or the knowledge may not be directly transferable to the design process. Evidence review has an important role in most approaches to intervention development [2] and formal knowledge in the form of meta-analysis and randomised controlled trials is viewed as the most robust form of evidence to inform decision making [39, 40]. However Dixon Woods highlights the risks of valuing scientific knowledge over practical and social wisdom: *“Scott sees part of the problem of [top down state] interventions as lying in hubris about the superiority of scientific knowledge and a corresponding under-valuing of insider*, *local*, *experience-based*, *contextual knowledge”* p96 [41, 42]. Our analysis suggests that intervention developers do value practical and social wisdom and that although evidence and theory are prioritised in published guidance, in practice, stakeholder opinion may be given more weight in design.

The ability of a design team to work effectively with different types of knowledge may also affect their capacity to generate innovative ideas. Nonaka identifies four ways in which explicit and tacit knowledge interact [16]. For intervention design a key point is that these different explicit and tacit knowledge interactions may not be equally successful in terms of generating novel ideas. For example, socialisation (tacit to tacit: individuals working together informally e.g. a co-design workshop) and internalisation (explicit to tacit: application of formal explicit knowledge through experience or experimentation e.g. engaging with prototypes) may be more likely to result in innovative ideas than externalisation (tacit to explicit e.g. conducting formal qualitative research) and combination (bringing together explicit knowledge e.g. producing a literature review) [43]. However, with any design process the openness to new ideas when engaging with people is likely to be critical, regardless of roles, the method of engagement or type of knowledge being discussed.

We identified that the extent to which creativity was discussed and the process of ideation was made explicit varied depending on the mode in which the designers were operating. Particularly among developers in the informed mode, there was a tendency to see design as being more about identifying components that had been found to be effective in previous research, rather than seeking to expand the possible solutions with more “blue sky” thinking. There is a risk with this approach to development of reinventing the wheel–endlessly recycling components of existing interventions. Two recent reviews of innovation in health care found that most developments they identified were adaptations or iterations rather than de-novo [20, 44]. This may be because more exploratory research does not sit easily with health research standards, and is seen as less methodologically rigorous and more risky [20]. Small and incremental changes can be impactful if, for example, they lead to substantially wider take up of an intervention, or successful application in new contexts. However, in the longer term, without more radical and disruptive interventions, the pace of improvement in health is likely to be slow [45].

Roberts et al [19] suggest that design thinking can help teams to avoid some of the pitfalls of other healthcare design processes including group think and a tendency to suppress dissenting voices [1]. They further suggest that there are important differences between design-led and science-led approaches with scientists more likely to use: “*pre-formed hypotheses or theory-driven solution approaches”*, while designers *“put more emphasis on synthesizing information and ideas from many different sources*, *in search of new and unconventional solutions*.*” P 12* [19]. Although our sample included examples where involvement of a designer or design thinking was felt to be hugely beneficial, it also included one example where the involvement of a designer had obstructed progress. Not all designers will work effectively with all problems and all teams. The differentiation between problem and solution focus can also be overstated. As in clinical decision making where the choice and process of diagnosis will be informed by the treatment options available [46], so with design, problem definition and design solution cannot be fully disentangled [17].

The six modes that we identified each had strengths and weaknesses in relation to the three areas of stability, working with knowledge and creativity. Current MRC guidance focuses on literature review and use of theory as key elements in the early phases of intervention development [6]. However our analysis suggests that those modes adhering most closely to this guidance–informed and structured design–may prioritise scientific knowledge over practical wisdom, and problem definition over solution identification, and as a result may experience equivocality and inadequate ideation.

We have described design as including ideation–the generating of ideas about the form, nature, components and delivery of the intervention, and decision making–deciding which of these ideas to include and which to reject. In our study the creative partnership mode incorporated both attention to ideation and to power sharing. Partnership approaches have tended to emphasise equality in decision making but may pay less attention to how to support diverse stakeholders to generate ideas [2]. Incorporating tools from design thinking when conducting co-design may assist diverse stakeholders to contribute fully to the design process–helping to address concerns expressed by some public contributors that their involvement is tokenistic and underdeveloped [47].

The design modes differed in the extent to which they involved different sectors–academic, health service, other non-profit sectors, for-profit organisations. In our sample, those who had delegated design, often across sectors, were positive about the benefits this brought in terms of expertise in design, and in preparing for implementation. However Horvath et al [25], found that developers of internet-based health interventions experienced challenges when working with sub-contractors, including when developing contracts and, because of differences in values, objectives and language. The negotiated design mode also involved working across sectors, typically involved translating a more abstract version of the intervention into a working version–with the negotiation being between a more academic development team and practitioners. Guidance on intervention development could usefully include attention to the benefits and challenges of working with design partners across sectors [7].

The “my-baby” example in our sample was a practising clinician. Intervention development for healthcare frequently happens outside an academic context [48]. Such development might be more open to methods and practices which originate from non-academic settings [20] Further research could usefully explore the extent to which such activity is different from that described here, so that best practice can be identified and shared.

Currently, we lack evidence as to which design modes for complex intervention development are most likely to lead to substantial health gains. Different modes will suit different intervention developments and it is likely that each mode has its place. However it is important that the pros and cons of each are understood so that developers can take steps to address potential gaps and shortcomings. Research that compares different approaches to intervention design would be helpful. Currently this is difficult not only because many design approaches are not standardised and not clearly described, but also because different fields have different ideas about how success of development should be measured [20, 32]. Clearer and more consistent reporting of the methods used in intervention development is needed.

The strengths of this study are the novelty of interviewing developers who differed in terms of their role within the development team their development experience, and the type of intervention and setting they had worked with. Design is a neglected area in healthcare research methodology, which could impact on both effectiveness of patient care and research waste [32, 49, 50]. The updated MRC framework [5] could usefully include approaches, such as co-creation and design thinking, which enable ideation and adequate attention to context, alongside evidence and theory.

A limitation of this paper is that the need to protect participants’ anonymity has restricted the information that we are able to provide about each interviewee and intervention development discussed. Anonymity was a significant concern for some participants, although others indicated that they would have been willing to have their quotations attributed to them as named individuals. Future research with experts might usefully consider a mixed consent model, whereby those participants willing to be named can be fully recognised for their contributions, and those preferring anonymity can participate on this basis [51]. A further limitation of this research was that the sample was predominantly UK based and did not include any participants from lower income countries. This was in part because the review of primary research from the wider INDEX study, used to identify participants, did not identify many papers from lower income countries. The small number of participants from North America and Europe reported similar issues in relation to design and we did observe considerable consistency in relation to our themes across very different developments. It therefore seems likely that the ideas discussed in this paper are relevant to many healthcare design contexts. We do not aim to create an exhaustive typology of design but rather to start a conversation about the way design differs in different contexts. There are likely to be other design modes and we would welcome further research to identify and characterise these, or to refine the modes that we suggest here. Publishing reports of design processes within intervention development [52] will assist this conversation.

## Conclusion

Health research has tended to neglect the design elements of intervention development, focusing more on the underpinning theory and evidence. This paper has highlighted the wide variation in approach to design taken by intervention developers. It has also highlighted that this is an aspect of intervention development that teams sometimes find challenging and where issues with equivocality (what are we doing and what do we do next) can be particularly apparent. Teams are starting to publish work describing in detail their design processes, providing helpful examples for other developers. However, as this literature expands there is a risk that novice developers will go from a lack of available models to drowning in a sea of different options. Future research should continue to bring together and critically examine different design approaches. Guidance for intervention development in health has tended to operate within a rational model that sees design as proceeding in a predictable and controlled manner from acquired knowledge. Our study suggests that design rarely reflects this rational ideal. Intervention developers require guidance and support to work effectively with different types of knowledge, to help design progress more smoothly and to maximise creativity.

## Supporting information

S1 File COREQ checklist. (DOCX)

## References

[1] Hoddinott P. A new era for intervention development studies. Pilot Feasibility Stud. 2015;1:36 10.1186/s40814-015-0032-0 27965814 PMC5153779

[2] O'Cathain A, Sworn K, Duncan E, Rousseau N, Turner K, Yardley L, et al Taxonomy of approaches to developing interventions to improve health: a systematic methods overview Pilot and Feasibility Studies. 2019;5(41). 10.1186/s40814-019-0425-6 PMC6419435 30923626

[3] O'Cathain A, Croot L, Duncan E, Rousseau N, Sworn K, Turner K, et al Guidance on how to develop complex interventions to improve health and healthcare. BMJ Open. 2019;9. 10.1136/bmjopen-2019-029954 PMC6701588 31420394

[4] Craig P, Dieppe P, Macintyre S, Michie S, Nazareth I, Petticrew M. Developing and evaluating complex interventions: the new Medical Research Council guidance. BMJ. 2008;337:a1655 10.1136/bmj.a1655 18824488 PMC2769032

[5] Craig P, Matthews L, Moore L, Simpson S, Skivington K. Updated Guidance: Developing and Evaluating Complex Interventions [DRAFT FOR CONSULTATION]. 2019.

[6] Craig P, Dieppe P, Macintyre S, Michie S, Nazareth I, Petticrew M. Developing and Evaluating Complex Interventions: New Guidance. Medical Research Council; 2008.

[7] Mummah S, Robinson T, King A, Gardner C, Sutton S. IDEAS (Integrate, Design, Assess, and Share): A Framework and Toolkit of Strategies for the Development of More Effective Digital Interventions to Change Health Behavior. Journal of Medical Internet Research. 2016;18(12):e317 10.2196/jmir.5927 27986647 PMC5203679

[8] Kushniruk A, Nøhr C. Participatory Design, User Involvement and Health IT Evaluation. Studies in health technology and informatics. 2016;222:139–51. 27198099

[9] Bessant J, Maher L. Developing radical service innovations in healthcare–the role of design methods. International Journal of Innovation Management. 2009;13(4):555–68.

[10] Spencer M, Dineen R, Phillips A. Co-producing services—Co-creating health. 2013.

[11] Band R, Bradbury K, Morton K, May C, Michie S, Mair F, et al Intervention planning for a digital intervention for self-management of hypertension: a theory, evidence and person-based approach. Implement Sci. 2017;12:13 10.1186/s13012-017-0546-3 28231840 PMC5324312

[12] O'Brien N, Heaven B, Teal G, Evans E, Cleland C, Moffatt S, et al Integrating Evidence From Systematic Reviews, Qualitative Research, and Expert Knowledge Using Co-Design Techniques to Develop a Web-Based Intervention for People in the Retirement Transition. Journal of Medical Internet Research. 2016;18(8):19. 10.2196/jmir.5790 PMC4989122 27489143

[13] Finch T, Bamford C, Deary V, Sabin N, Parry S. Making sense of a cognitive behavioural therapy intervention for fear of falling: qualitative study of intervention development. Bmc Health Services Research. 2014;14:436 10.1186/1472-6963-14-436 25252807 PMC4263069

[14] Koen P, Ajamian G, Burkart R, Clamen A, Davidson J, D'Amore R, et al Providing clarity and a common language to the "Fuzzy Front End". Res-Technol Manage. 2001;44(2):46–55.

[15] Smith P. Developing products in half the time: New rules new tools. New York: John Wiley & Sons; 1998.

[16] Nonaka I. A dynamic theory of organisational knowledge creation. Organ Sci. 1994;5(1):14–37.

[17] Ocker R. Influences on creativity in asynchronous virtual teams: A qualitative analysis of experimental teams. IEEE Trans Prof Commun. 2005;48(1):22–39.

[18] Cross N. Expertise in design: an overview. Design Stud. 2004;25(5):427–41.

[19] Roberts J, Fisher T, Trowbridge M, Bent C. A design thinking framework for healthcare management and innovation. HealthCare. 2016;4(1):11–4. 10.1016/j.hjdsi.2015.12.002 27001093

[20] Bazzano A, Martin J, Hicks E, Faughnan M, Murphy L. Human-centred design in global health: A scoping review of applications and contexts. PLoS One. 2017;12(11):24. 10.1371/journal.pone.0186744 PMC5665524 29091935

[21] Thies A. On the Value of Design Thinking for Innovation in Complex Contexts: A Case from Healthcare. Interact Des Archit. 2015(27):159–71.

[22] Norman D, Draper S. User-Centered System Design: New Perspectives on Human-Computer Interaction. Hillsdale, New Jersey: Lawrence Earlbaum Associates; 1986.

[23] IDEO. Human Centred Design Toolkit. San Francisco: IDEO; 2011.

[24] Hoddinott P, Pollock A, O'Cathain A, Boyer I, Taylor J, MacDonald C, et al How do researchers incorporate public, patient, clinician and staff perspectives into the design and conduct of their research. F1000Research [Internet]. 2018;7:752 10.12688/f1000research.15162.1 30364075 PMC6192439

[25] Horvath K, Ecklund A, Hunt S, Nelson T, Toomey T. Developing Internet-based health interventions: a guide for public health researchers and practitioners. J Med Internet Res. 2015;17(1):e28 10.2196/jmir.3770 25650702 PMC4319079

[26] O'Cathain A, Croot L, Duncan E, Rousseau N, Sworn K, Turner K, et al INDEX Study (IdentifyiNg and assessing different approaches to DEveloping compleX interventions) 2019 [Available from: https://www.sheffield.ac.uk/scharr/sections/hsr/mcru/indexstudy.

[27] Braun V, Clarke V. Using thematic analysis in psychology. Qualitative Research in Psychology. 2006;3(3):77–101.

[28] Saunders B, Sim J, Kingstone T, Baker S, Waterfield J, Bartlam B, et al Saturation in qualitative research: exploring its conceptualization and operationalization. Qual Quant. 2018;52(4):1893–907. 10.1007/s11135-017-0574-8 29937585 PMC5993836

[29] QSR. NVivo qualitative data analysis software. Version 11 ed: QSR International Pty Ltd; 2015.

[30] Michie S, Atkins L, West R. The Behaviour Change Wheel: A Guide to Designing Interventions Silverback Publishing; 2014.

[31] Murray E, Treweek S, Pope C, MacFarlane A, Ballini L, Dowrick C, et al Normalisation process theory: a framework for developing, evaluating and implementing complex interventions. BMC medicine. 2010;8:63 10.1186/1741-7015-8-63 20961442 PMC2978112

[32] Turner K, Rousseau N, Croot L, Duncan E, Yardley L, O’Cathain A, et al Understanding successful development of complex health and health care interventions and its drivers from the perspective of developers and wider stakeholders: an international qualitative interview study. BMJ Open [Internet]. 2019; 9:e028756 10.1136/bmjopen-2018-028756 31152042 PMC6549621

[33] Davidoff F, Dixon-Woods M, Leviton L, Michie S. Demystifying theory and its use in improvement. BMJ Qual Saf. 2015;24(3):228–38. 10.1136/bmjqs-2014-003627 25616279 PMC4345989

[34] Frishammar J, Floren H, Wincent J. Beyond Managing Uncertainty: Insights From Studying Equivocality in the Fuzzy Front End of Product and Process Innovation Projects. IEEE Trans Eng Manage. 2011;58(3):551–63.

[35] De Silva M, Breuer E, Lee L, Asher L, Chowdhary N, Lund C, et al Theory of Change: a theory-driven approach to enhance the Medical Research Council's framework for complex interventions. Trials. 2014;15:267 10.1186/1745-6215-15-267 24996765 PMC4227087

[36] Okhuysen G, Bechky B. Coordination in Organizations: An Integrative Perspective. Acad Manag Ann. 2009;3:463–502.

[37] Lowy I. The strength of loose concepts—boundary concepts, federative experimental stratefies and disciplinary growth—the case of immunology. Hist Sci. 1992;30(90):371–96.

[38] Visser W. More-or-less following a plan during design—opportunistic deviations in specification. International Journal of Man-Machine Studies. 1990;33(3):247–78.

[39] Murad M, Asi N, Alsawas M, Alahdab F. New evidence pyramid. Evidence-based medicine. 2016;21(4):125–7. 10.1136/ebmed-2016-110401 27339128 PMC4975798

[40] Grimshaw J, Russell I. Achieving health gain through clinical guidelines. I: Developing scientifically valid guidelines. Quality in health care: QHC. 1993;2(4):243–8. 10.1136/qshc.2.4.243 10132459 PMC1055154

[41] Dixon-Woods M. The problem of context in quality improvement. London; 2014.

[42] Scott J. Seeing like a state: How certain schemes to improve the human condition have failed. Yale University Press; 1998.

[43] Schulze A, Hoegl M. Organizational knowledge creation and the generation of new product ideas: A behavioral approach. Res Policy. 2008;37(10):1742–50.

[44] Lunze K, Higgins-Steele A, Simen-Kapeu A, Vesel L, Kim J, Dickson K. Innovative approaches for improving maternal and newborn health—A landscape analysis. BMC Pregnancy Childbirth. 2015;15:19 10.1186/s12884-015-0433-3 26679709 PMC4683742

[45] Christensen C, Bohmer R, Kenagy J. Will disruptive innovations cure health care?. Harv Bus Rev. 2000;78(5):102–112. 11143147

[46] Berg M. The construction of medical disposals Medical sociology and medical problem-solving in clinical practice. Sociol Health Ill. 1992;14(2):151–80.

[47] Ocloo J, Matthews R. From tokenism to empowerment: progressing patient and public involvement in healthcare improvement. BMJ Qual Saf. 2016;25(8):626–32. 10.1136/bmjqs-2015-004839 26993640 PMC4975844

[48] Casarett D, Karlawish J, Sugarman J. Determining when quality improvement initiatives should be considered research—Proposed criteria and potential implications. JAMA-J Am Med Assoc. 2000;283(17):2275–80. 10.1001/jama.283.17.2275 10807388

[49] Chalmers I, Glasziou P. Avoidable waste in the production and reporting of research evidence. Lancet. 2009;374(9683):86–9. 10.1016/S0140-6736(09)60329-9 19525005

[50] Chalmers I, Bracken M, Djulbegovic B, Garattini S, Grant J, Gulmezoglu A, et al How to increase value and reduce waste when research priorities are set. Lancet. 2014;383(9912):156–65. 10.1016/S0140-6736(13)62229-1 24411644

[51] Hawe P. Info sheet. Communicating Public Policy. Sydney: Menzies Centre for Health Policy, University of Sydney; 2017.

[52] Duncan E, O’Cathain A, Rousseau N, Croot L, Sworn K, Turner KM, et al Reporting GUIDance for intervEntion Development in health research (GUIDED) 2017 [Available from: http://www.equator-network.org/library/reporting-guidelines-under-development/reporting-guidelines-under-development-for-other-study-designs/#80. 10.1136/bmjopen-2019-033516 PMC7245409 32273313

