# Supplementary material for: Attending to design when developing complex health interventions: A qualitative interview study with intervention developers and associated stakeholders
Source: PLoS One. 2019 Oct 15;14(10):e0223615. doi: 10.1371/journal.pone.0223615 (PMC6793869; doi:10.1371/journal.pone.0223615)
Supplement: S1 File — (DOCX) [file pone.0223615.s001.docx]

**Consolidated criteria for reporting qualitative studies (COREQ): 32-item checklist**

| **No. Item** | **Guide questions/description** | **Reported on Page #** |
| --- | --- | --- |
| **Domain 1: Research team and reﬂexivity** |  |  |
| *Personal Characteristics* | ` |  |
| 1. Interviewer/facilitator | Which author/s conducted the interviews? | Page 6: NR conducted the interviews. |
| 2. Credentials | What were the researcher’s credentials? E.g. PhD, MD | PhD |
| 3. Occupation | What was their occupation at the time of the study? | Research Fellow. |
| 4. Gender | Was the researcher male or female? | Female. |
| 5. Experience and training | What experience or training did the researcher have? | Page 6: ‘All interviews were conducted by NR, an experienced qualitative researcher’ |
| *Relationship with participants* |  |  |
| 6. Relationship established | Was a relationship established prior to study commencement? | No relationship was established prior to the study but we explain in the paper that the interviewer familiarized herself with the participants’ published intervention development work so that she could ask relevant questions (page 6) . We also explain that some participants were well known within the research community (page 7). |
| 7. Participant knowledge of the interviewer | What did the participants know about the researcher? e.g. personal goals, reasons for doing the research | Participants were told that NR was the researcher employed on the study to conduct the qualitative work. If a participant asked during the interview about NRs background she explained that she was an experienced health services researcher but had more experience of evaluation and had done relatively little intervention development. For interviews with funders NR explained that she was also an advisor with the NIHR Research Design Service which supports applicants applying for research funding. |
| 8. Interviewer characteristics | What characteristics were reported about the interviewer/facilitator? e.g. Bias, assumptions, reasons and interests in the research topic | Page 7 states the background of those team members involved in data analysis including the interviewer. |
| **Domain 2: study design** |  |  |
| *Theoretical framework* |  |  |
| 9. Methodological orientation and Theory | What methodological orientation was stated to underpin the study? e.g. grounded theory, discourse analysis, ethnography, phenomenology, content analysis | This was a pragmatic study that was not underpinned by a specific methodological orientation or theory. It did, however, draw on some of the principles of grounded theory, in the sense that early data collection influenced some of the later interviews |
| *Participant selection* |  |  |
| 10. Sampling | How were participants selected? e.g. purposive, convenience, consecutive, snowball | We explain that potential participants through various routes, which are listed on page 6. We explain that sampling was purposive in terms of maximizing diversity according to criteria which we give on page 5 |
| 11. Method of approach | How were participants approached? e.g. face-to-face, telephone, mail, email | We explain potential participants were informed about the study by email (page 6). |
| 12. Sample size | How many participants were in the study? | 21 individuals were interviewed. This is stated in the abstract and on page 8. |
| 13. Non-participation | How many people refused to participate or dropped out? Reasons? | Page 8: we explain that ‘Twenty-nine individuals were invited for interview and 21 agreed (table 1). Most of those who declined to be interviewed were outside the UK.’. |
| *Setting* |  |  |
| 14. Setting of data collection | Where was the data collected? e.g. home, clinic, workplace | We explain on page 8 that seventeen of the interviews were conducted by telephone, two by Skype (with video), and two (both PPI contributors) on a face-to-face basis.’ |
| 15. Presence of non-participants | Was anyone else present besides the participants and researchers? | No. |
| 16. Description of sample | What are the important characteristics of the sample? e.g. demographic data, date | Dates of interviews (February 2017 to January 2018) and characteristics of the sample (gender, country, role in intervention development, discipline) are given on page 8 and in table 1. |
| *Data collection* |  |  |
| 17. Interview guide | Were questions, prompts, guides provided by the authors? Was it pilot tested? | We explain on page 6 a topic guide was used and outline the main areas covered. The guide was not pilot tested, but we explain that the topic guide evolved during data collection (page 7). |
| 18. Repeat interviews | Were repeat interviews carried out? If yes, how many? | No |
| 19. Audio/visual recording | Did the research use audio or visual recording to collect the data? | We explain on page 6 all the interviews were audio-recorded. |
| 20. Field notes | Were ﬁeld notes made during and/or after the interview or focus group? | We explain that NR made reflective field notes after each interview (page 6-7) |
| 21. Duration | What was the duration of the interviews or focus group? | Duration of the interviews is provided on page 8. |
| 22. Data saturation | Was data saturation discussed? | We explain on page 7 that data collection ended when data saturation has been reached. |
| 23. Transcripts returned | Were transcripts returned to participants for comment and/or correction? | Transcripts were not routinely returned but this was offered to participants. One individual did request to see the transcript of their interview and did change part of the text. |
| **Domain 3: analysis and ﬁndings** |  |  |
| *Data analysis* |  |  |
| 24. Number of data coders | How many data coders coded the data? | We explain on page 7, four researchers coded data, and that these four researchers and another member of the research team discussed emerging analysis at regular team meetings. |
| 25. Description of the coding tree | Did authors provide a description of the coding tree? | No. |
| 26. Derivation of themes | Were themes identiﬁed in advance or derived from the data? | Themes were derived from the data. This is indicated in our description of how the data were analysed. |
| 27. Software | What software, if applicable, was used to manage the data? | NVivo 11 was used to manage the data. This is stated on page 7. |
| 28. Participant checking | Did participants provide feedback on the ﬁndings? | No. |
| *Reporting* |  |  |
| 29. Quotations presented | Were participant quotations presented to illustrate the themes/ﬁndings? Was each quotation identiﬁed? e.g. participant number | Quotes have been used in the Results section. They have been tagged with the participants unique identification number. |
| 30. Data and ﬁndings consistent | Was there consistency between the data presented and the ﬁndings? | Yes |
| 31. Clarity of major themes | Were major themes clearly presented in the ﬁndings? | Yes |
| 32. Clarity of minor themes | Is there a description of diverse cases or discussion of minor themes? | We have indicated when a theme relates to just one individual e.g. the ‘my baby’ example and incorporated this case in to the broader analysis. We have taken care to look for disconfirming evidence in relation to minor as well as major themes – an example is that relating to designers and stabilization on page 19 |
